# Items

**Published:** 1902-04

**Authors:** 


### ITEMS.

The Charles Roome Parmele Company, manufacturing chemists of New York, proprietors and manufacturers of arsenauro, mercauro and other well-known preparations, have recently removed from 92 William Street and 36 Platt Street, to 45 John Street, New York, in order to secure more ample accommodations for their constantly increasing business.

---

The validity of the phenacetine patent has been sustained in the United States Circuit Court of Appeals for the third circuit, the court of last resort, in the case of Conrad D. Maurer vs. Farbenfabriken of Elberfeld Company. All infringers, the company now declares, will be prosecuted to the full extent of the law.

